# Supplementary material for: Genome-Wide Identification and Expression Analysis of BBX Transcription Factors in Iris germanica L
Source: Int J Mol Sci. 2021 Aug 17;22(16):8793. doi: 10.3390/ijms22168793 (PMC8396303; doi:10.3390/ijms22168793)
Supplement: Supplementary file 1 [file ijms-22-08793-s001.zip › ijms-1314176-supplementary.pdf]

International Journal of
Molecular Sciences

MDPI

Supplementary Materials:

a

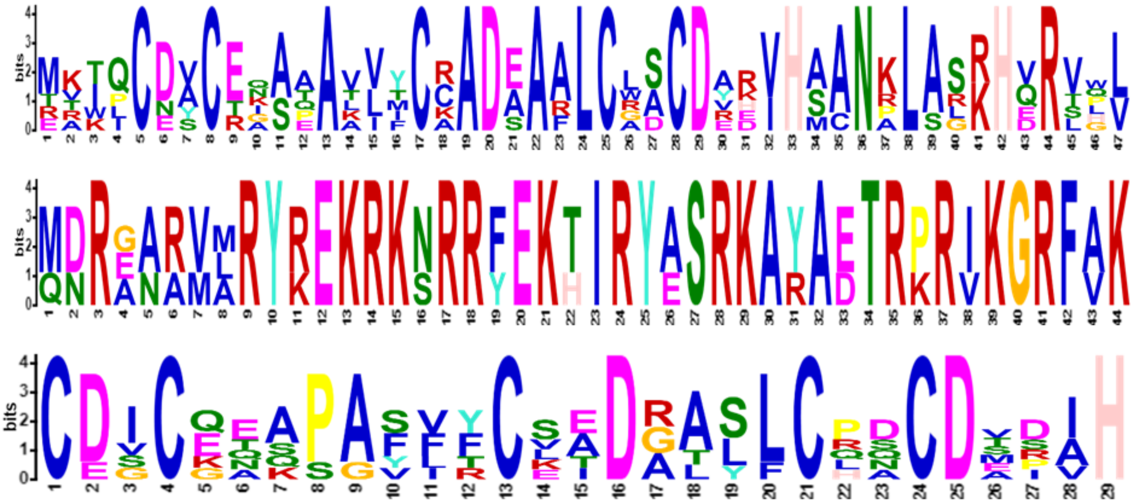

b

Name	Domains	B-BOX1	B-BOX2	CCT	Protein Structure
IgBBX1	2B-BOX+CCT	12-54	54-97	232-274	
IgBBX2	2B-BOX+CCT	22-64	64-104	253-296	
IgBBX3	2B-BOX	4-47	56-96		
IgBBX4	2B-BOX	5-45	63-106		
IgBBX5	2B-BOX	5-47	57-98		
IgBBX6	2B-BOX+CCT	14-57	57-100	423-465	

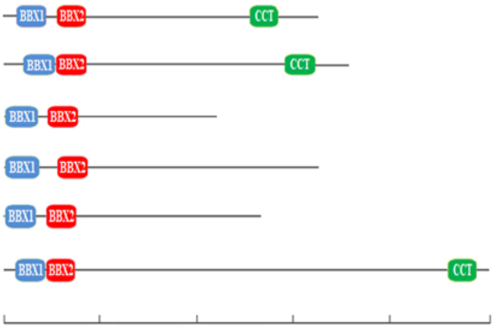

Figure S1. Conserved domains and structures of IgBBX proteins. (**a**) The conserved domains in the IgBBX proteins. (**b**) Structure of the IgBBX proteins. The scale bar represents 100 amino acids.

Table S1. PCR primer sequences utilized in this study

Primer Name	Sequence (5' to 3')	Annotation
**AP**	AAGCAGTGGTATCAACGCAGAGTAC	Universal primers for 3' RACE
**dT-AP**	AAGCAGTGGTATCAACGCAGAGTACTTTTTTTTTTTTTTTT	
**AUAP**	GGCCACGCGTCGACTAGTAC	Universal primers for 5' RACE
**AAP**	GGCCACGCGTCGACTAGTACGGGIIGGGIIGGGIIG	
**IgBBX1-F**	TTCTGGGGACTGTGCGCT	Amplify a fragment of the *IgBBXs*
**IgBBX1-R**	TGGCGAACCTTCCCTTGAT	
**IgBBX2-F**	GGGAGGAGGAGAGAGGAGCTA	

**IgBBX2-R**	CCTGTTCTTTCTCTTCTCCCTGTA	
**IgBBX3-F**	GTAGAGGAGAGTGTGTGGGCG	
**IgBBX3-R**	ATGTTAGGTCCAGGTTATTCCCA	
**IgBBX4-F**	GAGATGAAGATACAGTGCAACGC	
**IgBBX4-R**	GGTTCTCGTGGAAGTCGTAGG	
**IgBBX5-F**	GATACAATGTGATGCATGCGA	
**IgBBX5-R**	CATCATCGTCAGCAATCTCG	
**IgBBX6-F**	GTTCGCTGCTCCACCGAC	
**IgBBX6-R**	TTGACGAAACGGCCCTTTA	
**IgBBX1-ORF-F**	AACTCACTAATAATGGAGAAGGGC	ORF of *IgBBXs*
**IgBBX1-ORF-R**	CTCACTGGCAGTGGTCACTCA	
**IgBBX2-ORF-F**	ATGGCATCTAGAGAAGCAGAGGGA	
**IgBBX2-ORF-R**	TCAGAAGGAGGGGACAATTCCATAT	
**IgBBX3-ORF-F**	GAGAGAGTAGAGGAGAGTGTGTGG	
**IgBBX3-ORF-R**	CACATTACATTCTGAAGGGCTC	
**IgBBX4-ORF-F**	TACACACATAACGAAAGATAGGAAA	
**IgBBX4-ORF-R**	CGGACCGAGAAGTAGCCTAG	
**IgBBX5-ORF-F**	AGAGATGAAGATACAATGTGATGCA	
**IgBBX5-ORF-R**	ACGACATCGATATACATAATCATGC	
**IgBBX6-ORF-F**	TTTTGAGCACGGGAGCGA	
**IgBBX6-ORF-R**	CAGCAGAACTAGCCACCAGTCTC	
**UBC9-F**	CCGACAATCTCCCACAAACA	qRT-PCR for *IgBBXs*
**UBC9-R**	GATGATCCGTTGGTTCCAGAG	
**IgBBX1-RT-F**	TATAGCTCCTACGCTGCCAAT	
**IgBBX1-RT-R**	GTATCGTCTTCTCGAACCGC	
**IgBBX2-RT-F**	CAGCAGACCACTAAGCCAGTT	
**IgBBX2-RT-R**	ATTTGACACTTCAGCCATTGC	
**IgBBX3-RT-F**	AGGCAGAGGGTTGAGTTTCC	
**IgBBX3-RT-R**	TCGGTAAGGTCTCATGTTGAGAT	
**IgBBX4-RT-F**	AAGTAAGCAAGGACGGTAACCT	
**IgBBX4-RT-R**	GCCTACCACTATCCGCCTTA	
**IgBBX5-RT-F**	TGTCTGATCCTGAGTCATGTGAC	
**IgBBX5-RT-R**	ATCGTCAGCAATCTCGGTTC	
**IgBBX6-RT-F**	GGGATTTTAATCTTGGACGAAC	
**IgBBX6-RT-R**	AGGTCACTGTAACTCTTGATCGTAA	
**IgBBX1-EcoR I-F**	GGAATTCCAACTCACTAATAATGGAGAAGGGC	Vector construction of *IgBBXs*
**IgBBX1-BamH I-R**	CGGGATCCCGCTCACTGGCAGTGGTCACTCA	
**IgBBX2-EcoR I-F**	GGAATTCATGGCATCTAGAGAAGCAGAGGGA	
**IgBBX2-BamH I-R**	CGGGATCCCGTCAGAAGGAGGGGACAATTCCATAT	
**IgBBX3-EcoR I-F**	GGAATTCGAGAGAGTAGAGGAGAGTGTGTGG	
**IgBBX3-BamHI-R**	CGGGATCCCGCACATTACATTCTGAAGGGCTC	
**IgBBX4-EcoR I-F**	GGAATTCTACACACATAACGAAAGATAGGAAA	

**IgBBX4-BamH I-R**	CGGGATCCCGCGGACCGAGAAGTAGCCTAG
**IgBBX5-EcoR I-F**	GGAATTCAGAGATGAAGATACAATGTGATGCA
**IgBBX5-BamH I-R**	CGGGATCCCGACGACATCGATATACATAATCATGC
**IgBBX6-EcoR I-F**	GGAATTCTTTTGAGCACGGGAGCGA
**IgBBX6-Pst I-R**	GCTGCAGCCAGCAGAACTAGCCACCAGTCTC
